# Multiple genetic variations of chronic rhinosinusitis with nasal polyps are associated with respiratory parameters in men with obstructive sleep apnea

**DOI:** 10.1007/s11325-021-02356-6

**Published:** 2021-03-26

**Authors:** Qianqian Zhang, Xiaoting Wang, Xiangyu Cheng, Xiaolin Wu, Yunhai Feng, Huajun Xu, Huaming Zhu, Hongliang Yi, Weitian Zhang, Xinyi Li, Haibo Ye

**Affiliations:** 1 Department of Otolaryngology Head and Neck Surgery & Center of Sleep Medicine, Shanghai Jiao Tong University Affiliated Sixth People’s Hospital, 600 Yishan Road, Shanghai, 200233 People’s Republic of China; 2 Otolaryngology Institute of Shanghai Jiao Tong University, Yishan Road 600, Shanghai, 200233 China; 3 Shanghai Key Laboratory of Sleep Disordered Breathing, Shanghai, China; 4 The Central Laboratory of the Eighth People’s Hospital of Shanghai, Shanghai, China; 5 The Eighth People’s Hospital of Shanghai, Shanghai, China

**Keywords:** Chronic rhinosinusitis, Genetic risk score, Nasal polyps, Obstructive sleep apnea, Single nucleotide polymorphism

## Abstract

**Purpose:**

Patients with chronic rhinosinusitis with nasal polyps (CRSwNP) have a higher risk of obstructive sleep apnea (OSA). However, the relationship between CRSwNP and OSA remains unclear. The aim of this research study was to evaluate the association of multiple single nucleotide polymorphism (SNP) variations in CRSwNP with sleep- and breath-related parameters in men with OSA.

**Methods:**

We included eight CRSwNP SNPs in 2320 participants after strict screening. For each participant, the genetic risk score (GRS) was calculated based on the cumulative effect of multiple genetic variants of CRSwNP. A bivariate correlation analysis was used to assess the relationship between CRSwNP genetic polymorphisms and polysomnography parameters in men with OSA. Logistic regression analyses were used to assess the relationship between the risk of OSA and CRSwNP genetic polymorphisms.

**Results:**

In moderate OSA, rs28383314 was related to the oxygen desaturation index, and rs4807532 was positively associated with the microarousal index (*r* = 0.09, *P* = 0.03 and *r* = 0.11, *P* = 0.01, respectively). The CRSwNP GRS was positively correlated with the oxygen desaturation index and cumulative time percentage with SpO_2_ < 90% in moderate OSA (*r* = 0.13, *P* < 0.001 and *r* = 0.1, *P* = 0.01, respectively). There was no association between the CRSwNP GRS and the risk of OSA (OR = 1.007; 95% CI, 0.973–1.042; *P* = 0.702).

**Conclusion:**

In men with moderate OSA, single CRSwNP genetic variations correlated with sleep-related parameters, and the cumulative effects of CRSwNP genetic variations were associated with the hypoxic index. CRSwNP may be a predisposing condition for sleep disorders in men with moderate OSA.

**Supplementary Information:**

The online version contains supplementary material available at 10.1007/s11325-021-02356-6.

## Introduction

Obstructive sleep apnea (OSA) is a highly prevalent sleep disorder affecting 49.7% of men and 23.4% of women and is characterized by fragmented sleep, frequent episodes of upper airway collapse, and intermittent hypoxia [1, 2]. The pathophysiology of OSA is complicated and multifactorial, and a constricted upper airway during sleep is the most frequent observation [3]. Upper airway inflammation resulting in mucosal congestion may be an important mechanism for the development of OSA [4]. Sleep quality is known to be affected in individuals with chronic rhinosinusitis, and these patients have a higher risk of developing OSA [5, 6].

Chronic rhinosinusitis with nasal polyps (CRSwNP) is a phenotype of chronic inflammation of the paranasal and nasal sinus mucosa that partly or totally blocks the upper respiratory tract, and it correlates well with subjective nasal obstruction [7, 8]. Patients with CRSwNP complain of its negative effect on their quality of life and sleep, with reports of symptoms such as such as mouth breathing, sleep disturbance, and snoring being common [9, 10]. Research has suggested that impaired sleep quality is closely correlated with CRSwNP [11]. However, the aforementioned studies investigated the relationship between sleep quality and CRSwNP via subjective questionnaires, not objective data. Polysomnography (PSG) refers to a procedure carried out during sleep that is used to obtain physiologic parameters such as oxygen saturation, respiratory effort, and sleep stages to evaluate sleep-related breathing disorders, such as OSA. PSG can help record hypoxia- (breath) and sleep-related parameters in OSA [12]. Significant improvements in nasal resistance and a reduction in the apnea-hypopnea index (AHI) and apnea have been reported after polypectomy in patients with CRSwNP [8]. Another study also showed a reduction in the risk of OSA and an improvement in sleep quality after surgery [11]. These findings indicate a close relationship between OSA and CRSwNP; however, the inherent link between OSA and CRSwNP has not yet been elucidated.

The primary cause of CRSwNP and OSA remains unclear. Researchers have used many strategies to unravel the complex pathophysiology of CRSwNP and interactions of multiple risk factors [13]. Studying the genetic susceptibility to CRSwNP might be a valuable strategy to understand its pathogenesis. A genome-wide study of significant associations of CRSwNP using data from Iceland and the UK, including 4366 nasal polyps cases, 5608 chronic rhinosinusitis cases, and > 700,000 controls, identified 10 sequence variant loci with a risk of nasal polyps [14]. A familial genome-wide association study indicated that the genes *BICD2*, *VSIR*, *HLCS*, *HLA-DRA*, and *SLC5A1* are related to CRSwNP [15]. However, there are no current data regarding the influence of CRSwNP genetic variations on sleep- and breath-related parameters in patients with OSA. Here, we integrated single nucleotide polymorphisms (SNPs) of CRSwNP to identify the genetic variations of comorbidity in OSA and CRSwNP. As there is typically a tiny effect size of a single SNP on a disease phenotype, we pooled multiple genetic variants of CRSwNP using a cumulative effects model (genetic risk score, GRS) to explore the relationship between the CRSwNP genotype and OSA in a large-scale, cross-sectional cohort study. Notably, many studies have investigated sex differences in OSA and CRSwNP. The estimated prevalence of OSA has been shown to be higher in males than in females, and previous research has also identified a predominant sex distribution in males with CRSwNP [1, 16]. Therefore, male participants were recruited in the present study.

## Methods

### Participants

We enrolled participants suspected of having OSA between January 2008 and August 2017, defined according to PSG, in the ongoing Shanghai Sleep Heath Study, which collected anthropometric, genomic, and PSG data of the participants. The inclusion criteria were as follows: those aged > 18 years and who have not undergone any previous treatment. The exclusion criteria were as follows: (1) missing data on total SNPs > 15%; (2) other sleep disorders, such as narcolepsy or restless leg syndrome; and (3) missing data of cumulative time with SpO_2_ < 90% (CT90), sleep stage, and total sleep time (TST). Ultimately, 2320 participants were included in this study. The Ethics Committee of Shanghai Jiao Tong University Affiliated Sixth People’s Hospital approved this study, and informed consent was required and obtained from all participants.

### Anthropometric measurements

We measured the neck circumference (NC) at the level of cricothyroid membrane, waist circumference (WC) at the midway level between the iliac crest and the lowest costal margin, and hip circumference (HC) at the widest extension of the buttocks. The waist–hip ratio was calculated as the WC divided by HC. We calculated the body mass index by dividing the weight in kilograms by the height in meters squared (kg/m^2^). The diastolic and systolic blood pressure were measured at least three times using an automated electronic equipment (Omron Model HEM-752 Fuzzy, Omron Company), and the average reading was used for analysis.

### Polysomnographic evaluation and sleep- and breath-related parameters

Respiratory events and sleep parameters were obtained using PSG (Alice 5; Respironics Inc., Pittsburgh, PA, USA). Recordings including nasal and oral airflow, electrocardiogram, electroencephalogram, chin electromyogram, bilateral electro-oculogram, body posture, chest and abdominal movements, and finger pulse oximetry were collected during overnight sleep. Sleep stages and respiratory events were checked manually by skilled technicians according to the 2007 American Academic Sleep Medicine (AASM) criteria [17]. Hypopnea was identified as a ≥ 50% reduction in airflow accompanied by a decrease in oxygen desaturation ≥ 3%, and apnea was identified as the absence of airflow lasting for ≥ 10 s. The severity of OSA was determined based on the number of hypopnea and apnea events per hour during sleep (AHI). Non-OSA and mild, moderate, and severe OSA were defined as AHI < 5, 5–15, 15–30, and ≥ 30 events/h, respectively. The oxygen desaturation index (ODI) was calculated based on the number of events during overnight sleep in which oxygen desaturation was ≥ 3% per hour. The micro-arousal index was calculated as the number of arousals per hour of sleep. Non-rapid eye movement sleep was categorized into three stages: stage 1, stage 2, and stage 3 according to the 2007 AASM criteria. Stage 1 (S1 time) is the beginning of sleep, in which alpha waves disappear and theta waves appear. In stage 2 (S2 time), electroencephalographic recordings tend to show characteristic “sleep spindles” and “K-complexes.” In Stage 3 (S3 time), slow-wave sleep occurs. Rapid eye movement (REM) sleep is defined as when rapid eye movements; low-voltage, mixed-frequency brain wave activity; and muscular hypotonia or atonia are present [17, 18].

### SNP selection and GRS calculation

We enrolled all the CRSwNP SNPs that met the levels of genome-wide significance from a genome-wide association study [14], including 17 CRSwNP SNPs (rs1888909, rs78757963, rs146597587, rs149206763, rs34210653, rs1391371, rs1837253, rs1444782, rs17718444, rs338598, rs6543124, rs174535, rs1050152, rs8046011, rs28383314, rs62408225, and rs4807542). After screening the variants in our genomic database [19], rs17718444, rs1050152, rs78757963, rs146597587, rs149206763, rs34210653, and rs1391371 were ruled out because the call rates were < 95%. SNP rs174535 did not meet the Hardy–Weinberg equilibrium and was excluded. SNP rs1837253 was also excluded because the minor allele frequencies were < 1%. Finally, rs6543124, rs28383314, rs62408225, rs1888909, rs1444782, rs8046011, rs4807542, and rs338598 were included for the analyses.

We used the GRS as a proxy for multiple genetic variants. The CRSwNP GRSs were generated by multiplying the SNPs’ effect size (*ß*) with the total number of risk alleles for each individual [19, 20]. The effect size and risk alleles are listed in Table 2. The data regarding risk alleles have also been reported in a genome-wide association study [14].

### Statistical analysis

Data are expressed as means ± standard deviation, medians (interquartile ranges), or categorical, whenever appropriate. Differences in descriptive variables among the three groups were analyzed using the polynomial linear trend test for continuous variables, chi-square test, and one-way analysis of variance. A bivariate correlation analysis was used to explore the relationships between the SNPs, GRSs, and variables. Binary logistic regression analyses were used to evaluate the relationship between the GRS and risk of OSA. The Hardy–Weinberg equilibrium was tested using PLINK (http://zzz.bwh.harvard.edu/plink/data.shtml). All statistical analyses were performed using the SPSS 21.0 software (IBM Corp., Armonk, NY, USA). A two-tailed *P* value < 0.05 was considered statistically significant.

## Results

### Baseline characteristics

We enrolled a total of 359 participants without OSA, 572 participants with moderate OSA, and 1389 participants with severe OSA in this study. The characteristics of all the participants are listed in Table 1. Participants with OSA were more obese, more hypoxic, and needed more sleep time than participants without OSA (*P* for linear trend < 0.001). The CT90 were 0.0, 1.7, and 13.9 in the non-OSA, moderate OSA, and severe OSA groups, respectively (*P* < 0.001). The percentages of S1 time/TST were 15.2, 18.2, and 17.9% in the non-OSA, moderate OSA, and severe OSA groups, respectively (*P* = 0.008). The percentages of S3 time/TST in the non-OSA, moderate OSA, and severe OSA groups were 17.3, 15.5, and 13.7%, respectively. The percentages of REM/TST in the non-OSA, moderate OSA, and severe OSA groups were 12.5, 10.9, and 10.9%, respectively. S3 time/TST and REM/TST in patients with severe OSA were lower than those in participants without non-OSA (*P* = 0.038 and *P* < 0.001, respectively).

**Table 1:** Characteristics of the general population classified by the severity of OSA

Characteristic	Non-OSA (*N* = 359)	Moderate OSA (*N* = 572)	Severe OSA (*N* = 1389)	*P* for trend
Demographic				
Age, years	35 (29–43)	41 (34–51)	41 (34–50)	< 0.001
BMI, Kg/m2	24.5 (22.9–26.5)	26.1 (24.2–28.4)	27.8 (25.8–30.1)	< 0.001
NC, cm	38 (37–40)	40 (38–41)	41 (39–43)	< 0.001
WC, cm	90 (84–95)	95 (90–100)	100 (94–106)	< 0.001
HC, cm	98 (94–102)	100 (97–105)	103 (99–108)	< 0.001
WHR	0.92 (0.88–0.95)	0.95 (0.91–0.98)	0.96 (0.94–1.0)	< 0.001
ESS	6 (2–10)	7 (3–11)	10 (5–14)	< 0.001
SBP, mmHg	120 (113–130)	125 (116–135)	128 (120–139)	< 0.001
DBP, mmHg	78 (71–83)	80 (73–88)	82 (76–90)	< 0.001
Smoke (%)	74 (20.6%)	153 (26.7%)	329 (23.7%)	0.095
Drink (%)	166 (46.2%)	266 (46.5)	795 (57.2%)	< 0.001
Sleep apnea				
AHI	2.4 (1.2–3.5)	21.8 (18.1–25.8)	55.7 (44.0–67.9)	< 0.001
MAI	15 (10.1–23.9)	21.9 (14.2–30.3)	33.5 (20.1–33.5)	< 0.001
ODI	2.4 (1.3–3.8)	21.5 (17.1–21.5)	55.9 (42.5–69.8)	< 0.001
CT90	0 (0–0.068)	1.7 (0.5–4.2)	13.5 (4.4–29.2)	< 0.001
Minimum SaO_2_	92 (89–94)	82 (77–87)	71 (62–79)	< 0.001
TST (min)	396.9 (352–443.9)	406.7 (359.3–446.7)	423.9 (380.4–464.3)	< 0.001
S1(min)	61.5 (42–92)	72 (46.5–107.9)	72.5 (45–112)	< 0.001
S1/TST (%)	15.2 (10.5–23.8)	18.2 (11–27.6)	17.9 (11.1–26.7)	0.008
S2(min)	196.5 (152–241.5)	200.3 (156.5–245)	216.5 (161.5–265)	< 0.001
S2/TST (%)	50.8 (41.7–59.1)	51.6 (42.4–59)	53.2 (42–62.5)	0.206
S3(min)	63.5 (42.5–91)	59.5 (39.5–92.5)	56.5 (32.5–96.5)	0.812
S3/TST (%)	17.3 (11.2–24.5)	15.5 (10.3–23.4)	13.7 (7.9–22.3)	0.038
REM (min)	47.5 (30.5–47.5)	43.5 (28.5–61.88)	43.5 (29.2–62.5)	0.337
REM/TST (%)	12.5 (8.4–16.3)	10.9 (7.2–15.3)	10.9 (7.3–14.9)	< 0.001

The data are presented as means and standard deviation; skewed data are presented as the median (interquartile range), and categorical data as the number (percentage). Differences in the baseline characteristics among the four groups were examined using the polynomial linear trend test for continuous variables and the linear-by-linear association test for dichotomous variables. *BMI* body mass index, *NC* neck circumference, *WC* waist circumference, *HC* hip circumference, *WHR* waist/hip ratio, *AHI* apnea–hypopnea index, *MAI* microarousal index, *ODI* oxygen desaturation index, *SaO*_*2*_ oxygen saturation, *REM* rapid eye movement, *TST* total sleep time, *ESS* Epworth sleepiness scale, *SBP* systolic blood pressure, *DBP* diastolic blood pressure, *CT90* cumulative time percentage with SpO_2_ < 90%, *S1* stage 1 sleep of non-rapid eye movement, *S2* stage 2 sleep of non-rapid eye movement, *S3* stage 3 sleep of non-rapid eye movement

The basic characteristics of CRSwNP SNPs and the effect size (*ß*) of SNPs in the GRS construction are listed in Table 2. The minor allele distribution of SNP rs8046011 between the non-OSA and OSA groups was significantly different (*P* = 0.033).

**Table 2:** Genome-wide significant (*P* < 5 × 10^−8^) loci associated with CRSwNP

Loci	SNP	Chromosome	Position	MAF	Minor/major allele	Risk allele	*β*	*P* allele
IL18R1	rs6543124	2	102987459	10.98	A/T	T	− 0.139	0.9756
6p21	rs28383314	6	32587213	27.07	T/C	C	− 0.128	0.8494
BACH2	rs62408225	6	90956409	4.78	G/A	A	− 0.128	0.2867
IL33	rs1888909	9	6197392	4.136	T/C	T	0.293	0.3196
10p14	rs1444782	10	9058671	9.373	A/G	G	− 0.174	0.2314
16p13	rs8046011	16	11320662	44.7	G/A	A	0.140	0.03318
GPX4	rs4807542	19	1104078	10.23	A/G	G	− 0.174	0.682
CYP2S1	rs338598	19	41701528	49.26	A/C	C	− 0.151	0.9236

*P* allele is the difference of the allele distribution between the cases and control group

*MAF* minor allele frequency

*ß* the effect size of SNP

### Correlations between CRSwNP SNPs and respiratory events

The associations between SNPs and OSA-related parameters are listed in the Online Resource Supplemental Tables. Overall, rs8046011 was negatively associated with CT90 (*r* = − 0.05, *P* = 0.03), and rs62408225 was negatively associated with REM and REM/TST (*r* = − 0.058, *P* = 0.01 and *r* = − 0.06, *P* = 0.005, respectively) (Online Resource Table S1). In the non-OSA group, rs4807542 was negatively associated with REM/TST (*r* = − 0.011, *P* = 0.04) (Online Resource Table S2). In the moderate OSA group, rs28383314 was positively correlated with ODI, and rs4807532 was positively correlated with the microarousal index (*r* = 0.09, *P* = 0.03 and *r* = 0.11, *P* = 0.01, respectively) (Table 3). Moreover, rs28383314 was inversely correlated with S1 time (*r* = − 0.06, *P* = 0.005); rs62408225 was negatively associated with REM time (*r* = − 0.08, *P* = 0.004) and REM/TST (*r* = − 0.07, *P* = 0.01); rs1888909 was correlated with REM (*r* = 0.06, *P* = 0.03) and TST (*r* = − 0.07, *P* = 0.02); rs1444782 was negatively correlated with the microarousal index (*r* = − 0.06, *P* = 0.005); and rs338598 was positively correlated with S1/TST (*r* = 0.06, *P* = 0.03) (Online Resource Table S3).

**Table 3:** The association between SNPs and OSA-related parameters in moderate OSA patients

characteristics	rs6543124		rs28383314		rs62408225		rs1888909		rs1444782		rs8046011		rs4807542		rs338598	
	r	P	r	P	r	P	r	P	r	P	r	P	r	P	r	P
AHI	0.03	0.48	0.07	0.11	0.04	0.39	0.01	0.79	0.02	0.65	0.06	0.15	0.003	0.93	0.004	0.92
min SaO_2_	0.02	0.59	− 0.07	0.10	− 0.04	0.35	0.05	0.28	− 0.03	0.45	− 0.04	0.37	0.002	0.96	− 0.04	0.35
ODI	− 0.01	0.74	0.09	0.03	0.06	0.18	− 0.05	0.27	0.01	0.76	− 0.02	0.69	0.06	0.14	0.08	0.07
CT90	− 0.02	0.60	0.04	0.32	0.02	0.56	− 0.06	0.14	0.03	0.53	− 0.07	0.09	0.02	0.63	0.04	0.40
MAI	0.001	0.98	0.04	0.36	0.03	0.43	0.03	0.53	0.07	0.13	0.04	0.33	0.11	0.01	0.03	0.49
TST	− 0.004	0.93	0.02	0.68	0.02	0.56	− 0.06	0.17	− 0.03	0.47	0.01	0.85	− 0.01	0.76	0.05	0.28
S1 (min)	− 0.01	0.73	0.05	0.25	0.04	0.29	− 0.04	0.39	− 0.01	0.85	− 0.001	0.98	− 0.03	0.49	0.02	0.63
S1/TST (%)	− 0.02	0.60	0.05	0.19	0.05	0.23	− 0.02	0.57	− 0.01	0.82	− 0.01	0.76	− 0.04	0.37	-0.01	0.89
S2 (min)	0.04	0.37	− 0.003	0.95	0.02	0.67	− 0.01	0.84	0.02	0.64	0.01	0.76	− 0.02	0.71	0.06	0.18
S2/TST (%)	0.05	0.20	− 0.03	0.54	0.01	0.84	0.03	0.55	0.04	0.38	0.02	0.65	− 0.003	0.94	0.05	0.26
S3 (min)	− 0.01	0.80	0.001	0.98	− 0.04	0.40	0.001	0.98	− 0.04	0.38	− 0.02	0.61	0.03	0.54	− 0.02	0.66
S3/TST (%)	− 0.02	0.67	− 0.01	0.80	− 0.04	0.31	0.02	0.61	− 0.04	0.38	− 0.03	0.53	0.04	0.37	− 0.04	0.36
REM (min)	− 0.03	0.51	0.003	0.95	− 0.05	0.22	− 0.06	0.17	− 0.01	0.76	0.04	0.30	− 0.003	0.94	− 0.02	0.70
REM/TST (%)	− 0.03	0.51	− 0.004	0.92	− 0.06	0.15	− 0.04	0.37	− 0.004	0.92	0.04	0.30	− 0.003	0.94	− 0.04	0.33

*AHI* apnea–hypopnea index, *SaO*_*2*_ oxygen saturation; *ODI* oxygen desaturation index, *CT90* cumulative time percentage with SpO_2_ < 90%, *MAI* micro-arousal index, *REM* rapid eye movement, *S1* stage 1 sleep of non-rapid eye movement, *S2* stage 2 sleep of non-rapid eye movement, *S3* stage 3 sleep of non-rapid eye movement, *TST* total sleep time

### Correlations between CRSwNP GRSs and respiratory events

The median GRS of non-OSA was 2.803 (range, 0.192–5.42), median moderate OSA GRS was 2.612 (range, 0.192–5.42), and median severe OSA was 2.803 (range, 0.192–5.42), and they were not significantly different among the three groups (*P* = 0.248). The distribution of GRS across the groups is shown in Figure S1. The correlations between CRSwNP GRS and OSA parameters are shown in the Online Resource Table S4. We observed no relationship between the CRSwNP GRS and respiratory events across all the participants with non-OSA and severe OSA (Online Resource Table S4 and S5). However, in the moderate OSA group, the CRSwNP GRS was positively correlated with ODI and CT90 (*r* = 0.13, *P* < 0.001 and *r* = 0.1, *P* = 0.01, respectively) (Table 4). A higher CRSwNP GRS was not associated with the risk of moderate OSA (odds ratio [OR] = 1.007; 95% CI, 0.973–1.042; *P* =0.702). No associations were found even after adjusting for age and body mass index (OR = 1.004; 95% CI, 0.968–1.042; *P* = 0.812).

**Table 4:** Correlations between CRSwNP GRS with clinical characteristics in moderate OSA.

Characteristics	*r*	*p*
AHI	0.02	0.69
minimum SaO_2_	-0.06	0.18
ODI	0.13	< 0.001
CT90	0.10	0.01
MAI	0.06	0.14
S1 (min)	0.03	0.44
S1/TST	0.02	0.65
S2 (min)	0.04	0.38
S2/TST	0.02	0.62
S3 (min)	-0.01	0.79
S3/TST	-0.01	0.79
REM (min)	-0.02	0.56
REM/TST	-0.05	0.29
TST	0.04	0.40

*GRS* genetic risk score, *AHI* apnea–hypopnea index, *SaO*_2_ oxygen saturation, *ODI* oxygen desaturation index, *CT90* percentage of time with SaO_2_ < 90 %, *MAI* micro-arousal index, *REM* rapid eye movement, *S1* stage 1 sleep of non-rapid eye movement, *S2* stage 2 sleep of non-rapid eye movement, *S3* stage 3 sleep of non-rapid eye movement, *TST* total sleep time

## Discussion

In this study, we investigated the association between cumulative effects of CRSwNP-related loci and sleep- and breath-related parameters in Chinese men with OSA. We found that CRSwNP SNPs were correlated with sleep-related parameters in patients with OSA. Furthermore, cumulative genetic variants of CRSwNP SNPs negatively affected hypoxia indicators, CT90 and ODI, indicating that CRSwNP potentially contributes to sleep-related respiratory disorders at the genetic level.

Nasal obstruction and discharge are the most frequent symptoms of CRSwNP [16]. Nasal breathing increases ventilatory drive and nasal airway obstruction, which may decrease the quality of sleep [21]. Sleep disturbance is also a universal disease accompanied by CRSwNP [11]. In recent years, various studies have investigated the relationship between CRSwNP and sleep quality. Patients with CRSwNP have been shown to have worse sleep quality, but surgery can significantly improve sleep disturbance [22, 23]. A study on an adult population from France showed that sleep and quality of life were negatively associated with CRSwNP, and patients attributed moderate and severe sleep disturbance to nasal obstruction caused by CRSwNP [24]. Moderate-to-severe OSA is associated with more severe hypoxia, inflammation, and oxidative stress, and hypoxia caused by OSA can trigger inflammatory cytokine release, which contributes to upper airway inflammation [25, 26]. However, the association between CRSwNP and sleep disorder is still unclear, and nasal obstruction may not be the sole cause of sleep disorder in patients with CRSwNP. Several studies have explored the relationship between sleep-disordered breathing and nasal surgery and found that there is no significant improvement after nasal surgery. Nakata et al. reported that the number of apnea and hypopnea episodes per hour did not change significantly after nasal surgery; however, two different studies showed that surgery was useful in improving sleep quality, ameliorating sleep-disordered breathing, and lowering nasal resistance [27, 28]. Sleep quality is not only correlated with the AHI score but also associated with oxygen saturation, sleep architecture, and sleep efficiency. Therefore, our study aimed to explore the relationship between CRSwNP and hypoxia- and sleep-related parameters in men with OSA.

The mechanisms underlying the association between CRSwNP and OSA remain unknown. In the nasal epithelial cells of patients with CRSwNP, hypoxia-induced vascular endothelial growth factor has been shown to play a vital role in the early stages of polyp formation [29]. Moreover, under hypoxia, the expression of hypoxia-inducible factor-1α and survivin was upregulated in EoL-1 cells in patients with CRSwNP [30]. Hypoxia has been shown to trigger nasal polyposis in vitro, and hypoxia-inducible factor-1 inhibitors suppressed polypoid growth in a murine model [31]. Since these studies could not fully explain the relationship between CRSwNP and hypoxia, we tried to strengthen our understanding at the genetic level. Our results showed that patients with specific CRSwNP SNP variants were prone to having sleep-related breathing disorders by identifying specific gene mutations, which illustrated the potential association between CRSwNP and the development of hypoxia in OSA at the genetic level.

Previous studies have investigated the association between CRSwNP SNPs and other diseases. It has been reported that the interleukin-33 SNP rs1888909 is associated with an increased risk of hay fever in high linkage disequilibrium via interaction with rs928413 and rs1342326 [32]. The GPX4 SNP rs4807542 was reported to be associated with Kashin–Beck disease, biliary tract cancer, and preeclampsia in a Chinese Han population [33–35]. To date, few studies have investigated SNPs rs1444782, rs338598, rs6543124, rs8046011, rs28383314, and rs62408225. Our data indicated that rs8046011 was correlated with CT90, and cumulative CRSwNP genetic variates were positively correlated with ODI and CT90. In the moderate OSA group, rs28383314 and rs4807532 were correlated with ODI and the microarousal index, respectively. These findings were not observed in the other groups. These loci may play a vital role in the pathogenesis of CRSwNP and, therefore, need to be investigated further in the future. These findings also indicate that patients with CRSwNP may be more prone to moderate OSA than severe OSA. This assumption is consistent with the results from a previous study of ours and has been observed in our clinic. Nasal obstruction may be partially responsible for OSA in patients with CRSwNP. A more sophisticated environmental and genetic background should be considered in future studies.

The present study aimed to obtain high-quality results using a laboratory-based PSG and large sample size. Additionally, we used multiple SNPs in a GRS model to assess the correlations among genetic variants with sleep and breath events. However, several limitations should be noted. First, only eight SNPs were selected, which were based on the statistical significance of *P* values less than 10^−8^. The population specificity of the genetic background was not considered; the selected SNPs were those that were discovered in European populations and not in East Asian populations since there is a lack of genome-wide association studies exploring CRSwNP in East Asian populations, especially in Chinese people. Second, this was a cross-sectional study, making it impossible to determine a causal link between CRSwNP SNPs and OSA. Third, variations in mutation types such as frame shift, splice site mutation, translocation, insertion, and deletion were not considered. Additionally, the effect of lifestyle, environmental factors, and educational level as potential confounding factors was not considered. Despite these limitations, this is a novel study exploring the potential link between susceptibility genes for CRSwNP and OSA symptoms in a population of Chinese men. Further studies investigating these mechanisms are needed to better understand the association between CRSwNP and OSA.

## Conclusion

Multiple CRSwNP genetic variants were positively correlated with the hypoxic parameters, ODI and CT90, in moderate OSA. Single SNPs were related to the sleep index. This may provide a genetic explanation for the susceptibility of developing OSA in patients with CRSwNP.

## Supplementary information

ESM 1 (DOCX 116 kb)
