# Supplementary material for: Multiple genetic variations of chronic rhinosinusitis with nasal polyps are associated with respiratory parameters in men with obstructive sleep apnea
Source: Sleep Breath. 2021 Mar 26;26(1):57–65. doi: 10.1007/s11325-021-02356-6 (PMC8857115; doi:10.1007/s11325-021-02356-6)
Supplement: Supplementary file 1 — (DOCX 116 kb) [file 11325_2021_2356_MOESM1_ESM.docx]

**Multiple genetic variations of chronic rhinosinusitis with nasal polyps are associated with respiratory parameters in men of obstructive sleep apnea**

**Online resource supplemental tables and figure**

Authors:

Qianqian Zhang^1,2,3*^, MD; Xiaoting Wang^1,2,3*^, MD; Xiangyu Cheng^1,2,3*^, MD; Xiaolin Wu^4^, PhD; Yunhai Feng^5^, PhD; Huajun Xu^1,2,3^, MD, PhD; Huaming Zhu^1,2,3^, MD, PhD; Hongliang Yi^1,2,3^, PhD; Weitian Zhang^1,2,3^, PhD; Xinyi Li^1,2,3#^, PhD; Haibo Ye^1,2,3#^, MD, PhD

*These authors contributed equally to this paper.

Email addresses:

Qianqian Zhang: [zqq2018ky@163.com](mailto:zqq2018ky@163.com)

Xiaoting Wang: [xiaotingw95@163.com](mailto:xiaotingw95@163.com)

Xiangyu Cheng: [1031710992@qq.com](mailto:1031710992@qq.com)

Xiaolin Wu: [wuxiaolin999@hotmail.com](mailto:wuxiaolin999@hotmail.com)

Yunhai Feng: [fengyunhaient@sina.com](mailto:fengyunhaient@sina.com)

Huajun Xu: [sunnydayxu2010@163.com](mailto:sunnydayxu2010@163.com)

Huaming Zhu: [zhmtiger@126.com](mailto:zhmtiger@126.com)

Hongliang Yi: [yihongl@126.com](mailto:%20yihongl@126.com)

Weitian Zhang: [drzhangwt@163.com](mailto:drzhangwt@163.com)

Xinyi Li: [lixinyilixinyi123@163.com](mailto:lixinyilixinyi123@163.com)

Haibo Ye: [yehaibo_2012@163.com](mailto:yehaibo_2012@163.com)

Affiliations:

^1^ Department of Otolaryngology Head and Neck Surgery & Center of Sleep Medicine, Shanghai Jiao Tong University Affiliated Sixth People’s Hospital, Yishan Road 600, 200233 Shanghai, China

^2^ Otolaryngological Institute of Shanghai Jiao Tong University, Yishan Road 600, 200233 Shanghai, China

^3^ Shanghai Key Laboratory of Sleep Disordered Breathing

^4^ The Central Laboratory of the Eighth People's Hospital of Shanghai

^5^ The Eighth People's Hospital of Shanghai

# Corresponding author:

Xinyi Li, PhD (Department of Otorhinolaryngology-Head and Neck surgery, Shanghai Jiao Tong University Affiliated Sixth People’s Hospital, 600 Yishan Road, Shanghai, 200233, P. R. China; Tel: +86-2164834143; Fax:+86-2164834143, email: [lixinyilixinyi123@163.com](mailto:lixinyilixinyi123@163.com))

Haibo Ye MD, PhD (Department of Otorhinolaryngology-Head and Neck surgery, Shanghai Jiao Tong University Affiliated Sixth People’s Hospital, 600 Yishan Road, Shanghai, 200233, P. R. China; Tel: +86-2164834143; Fax: +86-2164834143, email: [yehaibo_2012@163.com](mailto:yehaibo_2012@163.com))

Table S1 The association between SNPs and OSA-related symptoms in general population.

| characteristics | rs6543124 | | rs28383314 | | rs62408225 | | rs1888909 | | rs1444782 | | rs8046011 | | rs4807542 | | rs338598 | |
| --- | --- | --- | --- | --- | --- | --- | --- | --- | --- | --- | --- | --- | --- | --- | --- | --- |
|  | r | P | r | P | r | P | r | P | r | P | r | P | r | P | r | P |
| AHI | 0.01 | 0.79 | 0.02 | 0.39 | 0.01 | 0.66 | 0.002 | 0.93 | 0.02 | 0.25 | -0.02 | 0.28 | -0.02 | 0.34 | 0.01 | 0.59 |
| min SaO_2_ | 0.004 | 0.86 | 9×10^-5^ | 1.00 | 0.02 | 0.43 | -0.02 | 0.39 | -0.03 | 0.18 | 0.01 | 0.56 | 0.02 | 0.42 | -0.01 | 0.58 |
| ODI | 0.01 | 0.77 | 0.02 | 0.43 | 0.002 | 0.92 | 0.003 | 0.88 | 0.02 | 0.40 | -0.03 | 0.21 | -0.01 | 0.79 | 0.01 | 0.61 |
| CT90 | 0.01 | 0.65 | -0.002 | 0.94 | 0.01 | 0.67 | 0.001 | 0.97 | 0.03 | 0.23 | -0.05 | **0.03** | -0.03 | 0.19 | -0.003 | 0.89 |
| MAI | 0.005 | 0.82 | 0.02 | 0.28 | 0.02 | 0.26 | 0.01 | 0.79 | 0.01 | 0.57 | -0.02 | 0.42 | -0.01 | 0.59 | 0.03 | 0.16 |
| S1(min) | -0.005 | 0.82 | 0.002 | 0.91 | 0.01 | 0.55 | 0.01 | 0.57 | 0.02 | 0.34 | -0.002 | 0.92 | 0.02 | 0.43 | 0.03 | 0.14 |
| S1/TST (%) | -0.004 | 0.84 | -1.5×10^-4^ | 0.99 | 0.01 | 0.51 | 8.8×10^-4^ | 0.97 | 0.01 | 0.47 | -4.6×10^-4^ | 0.98 | 0.02 | 0.45 | 0.03 | 0.18 |
| S2(min) | 0.01 | 0.48 | 0.01 | 0.73 | 0.003 | 0.90 | 0.004 | 0.86 | -0.003 | 0.88 | -0.01 | 0.71 | -0.02 | 0.45 | -0.01 | 0.54 |
| S2/TST (%) | 0.01 | 0.52 | -0.004 | 0.83 | 0.005 | 0.82 | -0.01 | 0.73 | -0.002 | 0.94 | 2.0×10^-4^ | 0.99 | -0.01 | 0.53 | -0.01 | 0.63 |
| S3 (min) | 0.01 | 0.69 | 0.01 | 0.59 | 1.1×10^-4^ | 1.00 | 0.01 | 0.50 | -0.005 | 0.81 | -0.004 | 0.86 | 0.02 | 0.40 | -0.01 | 0.56 |
| S3/TST (%) | 0.01 | 0.69 | 0.01 | 0.58 | 0.004 | 0.86 | 0.01 | 0.53 | -0.01 | 0.71 | -0.001 | 0.95 | 0.02 | 0.44 | -0.02 | 0.40 |
| REM (min) | -0.02 | 0.28 | 0.01 | 0.80 | -0.058 | **0.01** | 0.01 | 0.48 | -0.01 | 0.73 | 0.01 | 0.53 | -0.03 | 0.11 | 0.02 | 0.39 |
| REM/TST(%) | -0.03 | 0.22 | 0.004 | 0.84 | -0.06 | **0.005** | 0.007 | 0.75 | -0.01 | 0.57 | 0.02 | 0.30 | -0.04 | 0.06 | 0.02 | 0.48 |
| TST | 0.004 | 0.84 | 0.02 | 0.47 | -0.003 | 0.89 | 0.03 | 0.22 | 0.002 | 0.93 | -0.01 | 0.54 | -0.002 | 0.94 | 0.003 | 0.90 |

AHI, apnea–hypopnea index; SaO_2_ oxygen saturation; ODI, oxygen desaturation index; CT90, cumulative time percentage with SpO_2_ < 90%; MAI, micro-arousal index; REM, rapid eye movement; S1, stage 1 sleep of Non-rapid eye movement; S2, stage 2 sleep of Non-rapid eye movement; S3, stage 2 sleep of Non-rapid eye movement; TST, total sleep time.

Table S2 The association between SNPs and OSA-related symptoms in Non-OSA population.

| characteristics | rs6543124 | | rs28383314 | | rs62408225 | | rs1888909 | | rs1444782 | | rs8046011 | | rs4807542 | | rs338598 | |
| --- | --- | --- | --- | --- | --- | --- | --- | --- | --- | --- | --- | --- | --- | --- | --- | --- |
|  | r | P | r | P | r | P | r | P | r | P | r | P | r | P | r | P |
| AHI | 0.06 | 0.26 | 0.02 | 0.69 | 0.03 | 0.59 | 0.002 | 0.97 | 0.03 | 0.60 | -0.07 | 0.18 | -0.04 | 0.40 | -0.03 | 0.63 |
| min SaO_2_ | 0.01 | 0.85 | -0.02 | 0.70 | 0.04 | 0.41 | -0.05 | 0.31 | 0.05 | 0.36 | -0.02 | 0.67 | 0.03 | 0.55 | 0.04 | 0.43 |
| ODI | -0.005 | 0.93 | -0.01 | 0.89 | -0.04 | 0.50 | -0.01 | 0.81 | -0.03 | 0.54 | 0.01 | 0.87 | -0.03 | 0.51 | -0.01 | 0.83 |
| CT90 | 0.08 | 0.13 | -0.05 | 0.39 | -0.03 | 0.62 | 0.03 | 0.62 | -0.02 | 0.69 | -0.07 | 0.18 | -0.04 | 0.49 | -0.02 | 0.77 |
| MAI | -0.09 | 0.11 | 0.04 | 0.42 | -0.03 | 0.61 | 0.03 | 0.60 | -0.07 | 0.23 | -0.11 | 0.06 | 0.03 | 0.53 | 0.02 | 0.69 |
| S1(min) | -0.01 | 0.84 | -0.05 | 0.38 | -0.04 | 0.40 | 0.02 | 0.77 | 0.08 | 0.12 | -0.06 | 0.23 | 0.07 | 0.20 | -0.06 | 0.30 |
| S1/TST (%) | 0.02 | 0.66 | -0.04 | 0.50 | -0.06 | 0.30 | 0.01 | 0.92 | 0.08 | 0.13 | -0.06 | 0.27 | 0.08 | 0.15 | -0.06 | 0.28 |
| S2(min) | -0.07 | 0.17 | -0.02 | 0.73 | 0.07 | 0.21 | -0.003 | 0.96 | -0.01 | 0.83 | 0.02 | 0.72 | -0.02 | 0.70 | 0.02 | 0.69 |
| S2/TST (%) | -0.05 | 0.36 | 0.02 | 0.73 | 0.07 | 0.18 | -0.01 | 0.80 | -0.03 | 0.58 | 0.05 | 0.35 | 0.01 | 0.91 | 0.03 | 0.60 |
| S3 (min) | 0.02 | 0.67 | -0.03 | 0.55 | -0.04 | 0.49 | 0.05 | 0.31 | -0.04 | 0.41 | -0.04 | 0.48 | -0.03 | 0.58 | 0.01 | 0.88 |
| S3/TST (%) | 0.06 | 0.26 | -0.01 | 0.86 | -0.04 | 0.49 | 0.04 | 0.44 | -0.06 | 0.25 | -0.03 | 0.53 | -0.04 | 0.47 | -0.01 | 0.89 |
| REM (min) | -0.05 | 0.34 | 0.02 | 0.74 | 0.01 | 0.92 | -0.04 | 0.50 | 0.05 | 0.39 | 0.06 | 0.28 | -0.10 | 0.05 | 0.07 | 0.19 |
| REM/TST(%) | -0.03 | 0.57 | 0.05 | 0.34 | -0.008 | 0.88 | -0.06 | 0.27 | 0.04 | 0.44 | 0.07 | 0.19 | -0.11 | **0.04** | 0.07 | 0.21 |
| TST | -0.07 | 0.16 | -0.06 | 0.27 | 0.01 | 0.80 | 0.03 | 0.56 | 0.03 | 0.60 | -0.03 | 0.58 | -0.03 | 0.62 | 0.01 | 0.79 |

AHI, apnea–hypopnea index; SaO_2_ oxygen saturation; ODI, oxygen desaturation index; CT90, cumulative time percentage with SpO_2_ < 90%; MAI, micro-arousal index; REM, rapid eye movement; S1, stage 1 sleep of Non-rapid eye movement; S2, stage 2 sleep of Non-rapid eye movement; S3, stage 2 sleep of Non-rapid eye movement; TST, total sleep time.

Table S3 The associations between SNPs and OSA-related symptoms in Severe-OSA population.

| characteristics | rs6543124 | | rs28383314 | | rs62408225 | | rs1888909 | | rs1444782 | | rs8046011 | | rs4807542 | | rs338598 | |
| --- | --- | --- | --- | --- | --- | --- | --- | --- | --- | --- | --- | --- | --- | --- | --- | --- |
|  | r | P | r | P | r | P | r | P | r | P | r | P | r | P | r | P |
| AHI | -0.04 | 0.13 | 0.02 | 0.52 | 0.01 | 0.60 | 0.01 | 0.65 | -0.001 | 0.96 | 0.01 | 0.61 | 0.004 | 0.87 | -0.002 | 0.95 |
| min SaO_2_ | 0.02 | 0.40 | 0.03 | 0.20 | 0.04 | 0.15 | -0.04 | 0.11 | -0.01 | 0.60 | -0.001 | 0.96 | 0.003 | 0.91 | 0.005 | 0.86 |
| ODI | -0.03 | 0.33 | 0.004 | 0.88 | -0.01 | 0.84 | 0.03 | 0.34 | -0.01 | 0.75 | 0.01 | 0.83 | 0.02 | 0.36 | -0.01 | 0.60 |
| CT90 | -0.01 | 0.78 | -0.01 | 0.62 | 0.01 | 0.72 | 0.02 | 0.48 | 0.02 | 0.53 | -0.03 | 0.31 | -0.02 | 0.46 | -0.02 | 0.54 |
| MAI | 0.01 | 0.69 | 0.02 | 0.56 | 0.03 | 0.25 | -4.4×10^-4^ | 0.84 | -0.06 | **0.005** | 0.007 | 0.75 | -0.01 | 0.57 | 0.03 | 0.27 |
| S1(min) | -3.2×10^-5^ | 0.84 | -0.06 | **0.005** | 0.007 | 0.75 | -0.01 | 0.57 | 0.01 | 0.58 | 0.02 | 0.57 | 0.02 | 0.35 | 0.05 | 0.06 |
| S1/TST (%) | -0.003 | 0.91 | -0.02 | 0.55 | 0.02 | 0.56 | 0.01 | 0.77 | 0.01 | 0.74 | 0.02 | 0.46 | 0.02 | 0.37 | 0.06 | **0.03** |
| S2(min) | 0.02 | 0.41 | 0.02 | 0.57 | -0.02 | 0.50 | 0.01 | 0.64 | -0.01 | 0.60 | -0.02 | 0.57 | -0.01 | 0.72 | -0.05 | 0.07 |
| S2/TST (%) | 0.01 | 0.70 | -0.001 | 0.96 | -0.01 | 0.65 | -0.02 | 0.49 | -0.01 | 0.64 | -0.02 | 0.54 | -0.02 | 0.49 | -0.04 | 0.13 |
| S3 (min) | 0.01 | 0.66 | 0.02 | 0.38 | 0.02 | 0.46 | 0.01 | 0.65 | 0.01 | 0.62 | 0.01 | 0.72 | 0.02 | 0.37 | -0.01 | 0.61 |
| S3/TST (%) | 0.01 | 0.79 | 0.02 | 0.37 | 0.03 | 0.32 | 0.01 | 0.84 | 0.01 | 0.62 | 0.01 | 0.65 | 0.02 | 0.50 | -0.01 | 0.67 |
| REM (min) | -0.02 | 0.58 | 0.004 | 0.90 | -0.08 | **0.004** | 0.06 | **0.03** | -0.02 | 0.57 | -0.01 | 0.71 | -0.03 | 0.29 | 0.02 | 0.45 |
| REM/TST(%) | -0.02 | 0.40 | -0.002 | 0.93 | -0.07 | **0.01** | 0.04 | 0.10 | -0.02 | 0.40 | -0.003 | 0.90 | -0.40 | 0.15 | 0.03 | 0.31 |
| TST | 0.02 | 0.38 | 0.03 | 0.23 | -0.02 | 0.47 | -0.07 | **0.02** | 0.002 | 0.95 | -0.007 | 0.80 | 0.02 | 0.51 | -0.02 | 0.41 |

AHI, apnea–hypopnea index; SaO_2_ oxygen saturation; ODI, oxygen desaturation index; CT90, cumulative time percentage with SpO_2_ < 90%; MAI, micro-arousal index; REM, rapid eye movement; S1, stage 1 sleep of Non-rapid eye movement; S2, stage 2 sleep of Non-rapid eye movement; S3, stage 2 sleep of Non-rapid eye movement; TST, total sleep time

Table S4 Linear regression of CRS GRS with clinical characteristics in Non-OSA population.

| characteristics | r | p |
| --- | --- | --- |
| AHI | 0.04 | 0.40 |
| minimum SaO_2_ | 0.08 | 0.15 |
| ODI | -0.04 | 0.50 |
| CT90 | 0.001 | 0.98 |
| MAI | 0.03 | 0.62 |
| S1(min) | 0.02 | 0.74 |
| S1/TST | 0.03 | 0.56 |
| S2(min) | -0.02 | 0.66 |
| S2/TST | -0.01 | 0.88 |
| S3(min) | -0.03 | 0.58 |
| S3/TST | -0.02 | 0.65 |
| REM (min) | -0.01 | 0.87 |
| REM/TST | 0.002 | 0.97 |
| TST | -0.03 | 0.58 |

GRS: genetic risk score; AHI, apnea–hypopnea index; SaO_2_ oxygen saturation; ODI, oxygen desaturation index; CT90, percentage of time with SaO_2_ <90 %, MAI, micro-arousal index; REM, rapid eye movement; S1, stage 1 sleep of Non-rapid eye movement; S2, stage 2 sleep of Non-rapid eye movement; S3, stage 2 sleep of Non-rapid eye movement; TST, total sleep time.

Table S5 Linear regression of CRS GRS with clinical characteristics in Severe-OSA population.

| characteristics | r | p |
| --- | --- | --- |
| AHI | -0.01 | 0.63 |
| minimum SaO_2_ | 0.04 | 0.13 |
| ODI | -0.02 | 0.45 |
| CT90 | -0.01 | 0.76 |
| MAI | 0.01 | 0.66 |
| S1(min) | 0.02 | 0.47 |
| S1/TST | 0.02 | 0.40 |
| S2(min) | -0.02 | 0.47 |
| S2/TST | -0.02 | 0.57 |
| S3(min) | 0.01 | 0.64 |
| S3/TST | 0.01 | 0.62 |
| REM (min) | -0.04 | 0.16 |
| REM/TST | -0.05 | 0.29 |
| TST | -0.01 | 0.73 |

GRS: genetic risk score; AHI, apnea–hypopnea index; SaO_2_ oxygen saturation; ODI, oxygen desaturation index; CT90, percentage of time with SaO_2_ <90 %, MAI, micro-arousal index; REM, rapid eye movement; S1, stage 1 sleep of Non-rapid eye movement; S2, stage 2 sleep of Non-rapid eye movement; S3, stage 2 sleep of Non-rapid eye movement; TST, total sleep time.

**Figure S1** The distribution of genetic risk score in different groups of OSA.

**
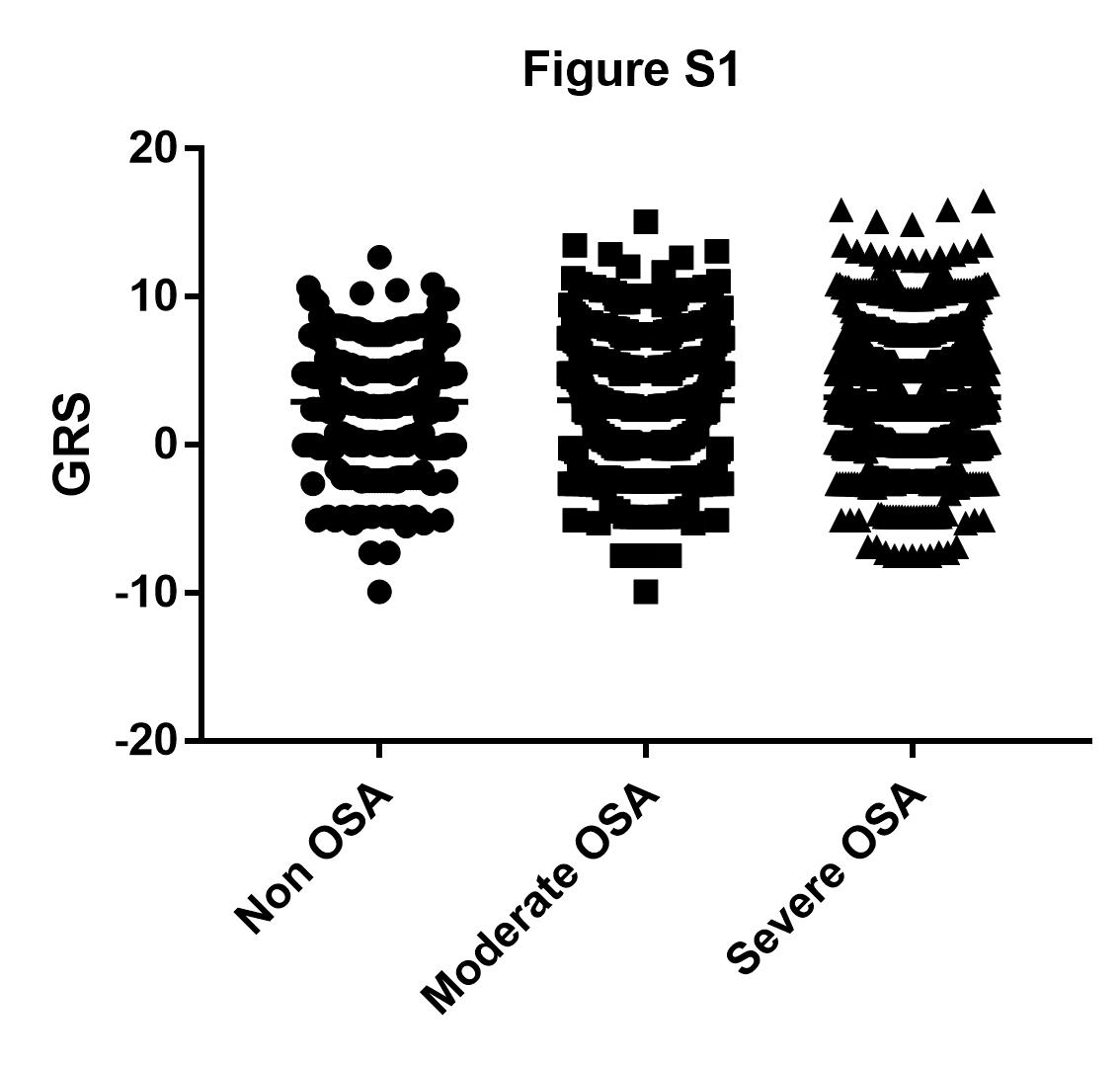
**
